# A Case of Multicentric Isocitrate Dehydrogenase-Wildtype Glioblastoma With Divergent Clonal Evolution

**DOI:** 10.7759/cureus.98066

**Published:** 2025-11-29

**Authors:** Nozomi Shibuya, Nayuta Higa, Toshiaki Akahane, Hajime Yonezawa, Mari Kirishima, Akihide Tanimoto, Ryosuke Hanaya

**Affiliations:** 1 Department of Neurosurgery, Kagoshima University Graduate School of Medical and Dental Sciences, Kagoshima, JPN; 2 Department of Pathology, Kagoshima University Graduate School of Medical and Dental Sciences, Kagoshima, JPN

**Keywords:** braf v600e mutation, divergent clonal evolution, egfr amplification, idh-wildtype glioblastoma, multicentric glioblastoma

## Abstract

Here, we present a rare case of multicentric glioblastoma in which two spatially distinct tumors exhibited different molecular profiles. A 56-year-old woman presented with progressive headache and speech difficulties. Magnetic resonance imaging (MRI) of the brain revealed two separate contrast-enhancing lesions, one in the left superior temporal gyrus and the other in the right middle temporal gyrus, with no connecting tract or edema on fluid-attenuated inversion recovery (FLAIR) imaging. Multicentric glioblastoma was suspected, and the patient underwent two-stage surgical resection of both tumors to achieve gross total removal. Histopathology confirmed both lesions as isocitrate dehydrogenase (IDH)-wildtype glioblastoma (World Health Organization (WHO) Grade 4), with typical features including extensive necrosis and microvascular proliferation. However, targeted next-generation sequencing revealed distinct genetic alterations in the two tumors. The left temporal lobe lesion harbored TERT promoter (TERTp) and BRAF p.Val600Glu (V600E) mutations. In contrast, the right temporal lobe lesion harbored a TERTp mutation, epidermal growth factor receptor (EGFR) amplification, MDM4 amplification, and a homozygous deletion of CDKN2A/B. The patient underwent standard postoperative chemoradiotherapy. One year after surgery, the right tumor recurred, whereas no recurrence was observed in the left temporal lobe. The BRAF p.Val600Glu (V600E) mutation in the left-sided tumor is a potential target for molecular therapy and may have contributed to the lack of recurrence. Multicentric glioblastomas are extremely rare, and only a few cases have been genetically profiled to date. Notably, most reported multicentric glioblastomas exhibit monoclonal driver mutations across lesions, suggesting a single origin. In the present case, the two tumors had different molecular signatures. This case underscores the importance of comprehensive genetic analysis of multicentric glioblastomas and highlights how divergent molecular pathologies can inform the prognosis and open opportunities for targeted treatment.

## Introduction

Glioblastoma is a World Health Organization (WHO) grade 4 malignant brain tumor with an extremely poor prognosis. Even with standard multimodal therapy, the median survival is only 15-20 months [1,2]. Glioblastomas (GBMs) are classified as isocitrate dehydrogenase (IDH)-wildtype, a molecular subtype defined by the absence of isocitrate dehydrogenase (IDH) mutations. IDH-wildtype glioblastomas typically occur in older adults, exhibit aggressive biological behavior, and frequently harbor genetic alterations, such as TERT promoter (TERTp) mutations and epidermal growth factor receptor (EGFR) amplification. In contrast, BRAF mutations are relatively uncommon in IDH-wildtype glioblastomas. Although most glioblastomas are solitary tumors, a subset of cases involves multiple lesions at diagnosis. The incidence of multifocal glioblastoma has been reported to range from around 0.5% up to 35% of cases [3-5]. These multiple lesions are further classified as multifocal glioblastomas, in which lesions are within the same brain region or lobe and may have contiguous spread on imaging, or as multicentric glioblastomas, in which lesions occur in different lobes or hemispheres with no direct continuity on imaging. True multicentric glioblastomas are relatively uncommon, accounting for approximately 2-5% of all glioblastoma cases [5]. Multicentric glioblastomas have a poor prognosis. Such patients often cannot undergo complete resection of all tumor sites and may respond poorly to conventional therapies [4]. Some studies have reported significantly worse survival in patients with multifocal or multicentric glioblastomas than in those with solitary tumors [4], although other analyses have found no clear survival difference [6]. Owing to the rarity of multicentric cases and the difficulty in treating and sampling multiple tumors, the molecular characteristics of multicentric glioblastomas remain poorly understood. It is unclear whether multiple lesions in one patient arise from a single precursor clone that has spread or whether they represent separate tumors that developed independently. Here, we report a case of multicentric glioblastoma in which two spatially separated lesions had distinct molecular profiles, suggesting divergent clonal evolution.

## Case presentation

**Figure 1:**
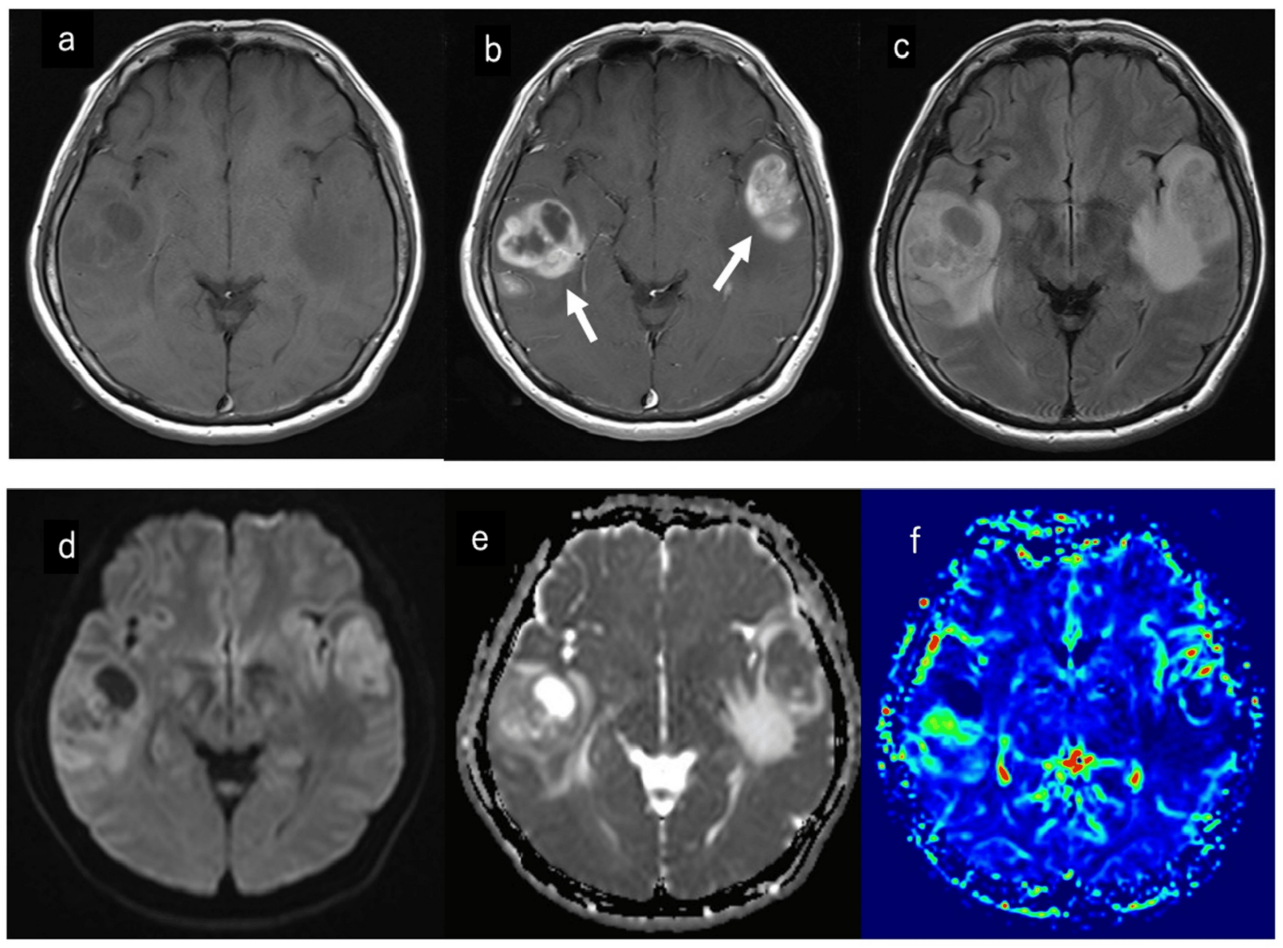
Preoperative brain MRI scans. (a) Preoperative T1-weighted MRI (axial). (b) Preoperative gadolinium-enhanced T1-weighted MRI, with white arrows indicating the tumors. (c) Preoperative FLAIR image (axial). (d) Preoperative DWI (axial). (e) Preoperative ADC image (axial). (f) Preoperative rCBV image (axial). FLAIR: fluid-attenuated inversion recovery; rCBV: relative cerebral blood volume; DWI: diffusion-weighted imaging; MRI: magnetic resonance imaging.

**Figure 2:**
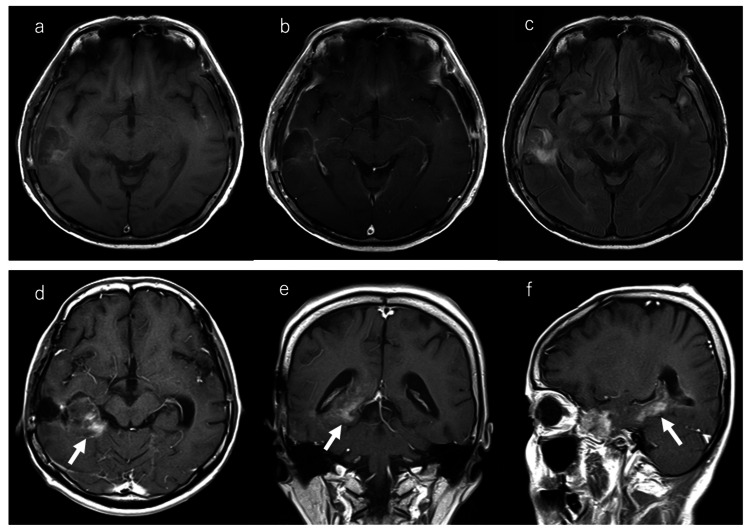
Postoperative MRI and recurrence MRI. (a) Postoperative T1-weighted MRI (axial). (b) Postoperative contrast-enhanced T1-weighted MRI. (c) Postoperative FLAIR image (axial). (d) Axial contrast-enhanced T1-weighted MRI at recurrence revealing a new ring-enhancing mass at the periphery of the right temporal resection cavity (white arrow). (e) Coronal contrast-enhanced T1-weighted MRI demonstrating the recurrent tumor (white arrow). (f) Sagittal contrast-enhanced T1-weighted MRI showing the recurrent tumor (white arrow). FLAIR: fluid-attenuated inversion recovery; MRI: magnetic resonance imaging.

A 56-year-old woman initially presented to a local physician with progressive speech difficulty and worsening headache, with a Karnofsky Performance Status (KPS) score of 80 at initial evaluation [7]. Magnetic resonance imaging (MRI) of the brain revealed two distinct intracranial lesions: one in the left superior temporal gyrus and the other in the right middle temporal gyrus. The left temporal lesion measured 31 × 20 × 27 mm, and the right temporal lesion measured 35 × 35 × 28 mm. The lesions appeared isointense to hypointense on T1-weighted images (Figure 1a) and showed ring-shaped enhancement with gadolinium on contrast-enhanced T1-weighted sequences (Figure 1b). Notably, there was no contiguous edema or connecting tract between the two tumors on fluid-attenuated inversion recovery (FLAIR) images and diffusion-weighted imaging (DWI) (Figures 1c, 1d), consistent with a multicentric presentation. Quantitative MRI analysis showed a mean apparent diffusion coefficient (ADC) value of 1.610 × 10⁻³ mm²/s for the right lesion and 1.098 × 10⁻³ mm²/s for the left lesion (Figure 1e). Perfusion imaging demonstrated a mean relative cerebral blood volume (rCBV) ratio (tumor/white matter) of 2.09 for the right lesion and 3.44 for the left lesion (Figure 1f). Based on these imaging findings, a multicentric glioblastoma was suspected. Following the onset of symptoms, the patient underwent the first stage of tumor removal 10 days later as part of a staged surgical approach. First, a craniotomy was performed to resect the left temporal lesion. Two days later, a second craniotomy was performed for resection of the right temporal lesion. Gross total resection was performed for both tumors. Postoperative MRI confirmed the absence of any residual enhancing tumors (Figures 2a-2c). 

**Figure 3:**
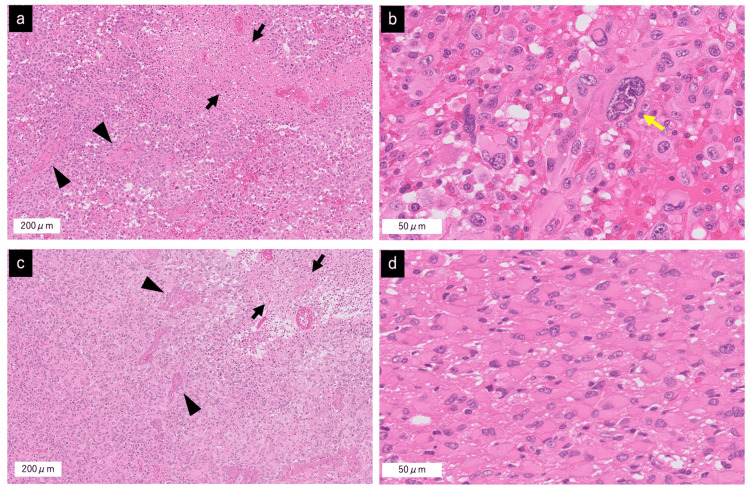
Histopathology of the left and right temporal lobe lesions. (a) Hematoxylin and eosin (H&E) stained section at low magnification, indicating high cellularity of tumor cells with palisading necrosis (black arrow) and microvascular proliferation (black arrowhead) at the left temporal lesion. (b) H&E section at higher magnification showing pleomorphic tumor cells occasionally containing giant cells with markedly enlarged bizarre nuclei (yellow arrow) at the left temporal lesion. (c) H&E at low magnification, showing a high cellularity of tumor cells with palisading necrosis (black arrow) and microvascular proliferation (black arrowhead) at the right temporal lesion. (d) H&E at high magnification showing gemistocytic tumor cells at the right temporal lesion.

Histopathological examination of both lesions confirmed a diagnosis of glioblastoma (IDH-wildtype). Each tumor showed pseudopalisading necrosis and microvascular proliferation, with tumor cells containing markedly atypical, enlarged nuclei. The left temporal lobe tumor demonstrated greater cellular pleomorphism with occasional giant tumor cells with bizarre nuclei (Figures 3a, 3b), compared to the right side, which contained gemistocytic tumor cells (Figures 3c, 3d). The proliferation index (MIB-1 labeling) was approximately 43% in the left lesion and 40% in the right lesion, indicating high proliferative activity in both lesions.

Comprehensive genetic analysis was performed for each tumor using a glioma-tailored next-generation sequencing panel [8]. These results reveal that the two lesions have different molecular profiles. The left temporal lobe tumor harbored a TERT promoter (TERTp) (variant allele frequency (VAFs): 24.4%) and a BRAF p.Val600Glu (V600E) mutation (VAFs: 17.0%). In contrast, the right temporal lobe tumor harbored a TERTp mutation (VAFs: 41.6%) along with amplification of the EGFR and MDM4 genes and a homozygous deletion of CDKN2A/B. These findings indicate that, despite similar histopathologies, the two glioblastomas had distinct genetic aberrations.

Postoperative therapy included the standard Stupp protocol for patients with glioblastoma. The patient underwent concurrent chemoradiation with temozolomide (TMZ) (75 mg/m^2^/day during radiotherapy) and focal brain irradiation (60 Gy in 30 fractions via intensity-modulated radiotherapy). Treatment was completed without significant adverse effects, and the patient was discharged with a KPS of 100. Adjuvant maintenance TMZ chemotherapy was subsequently administered along with bevacizumab and tumor-treating field therapy as part of a multidisciplinary regimen.

At the one-year postoperative follow-up, surveillance MRI revealed tumor recurrence at the margin of the right temporal lobe resection cavity, whereas no recurrence was observed at the left temporal lobe resection site (Figures 2d-2f). At the time of recurrence, the patient's KPS had declined to 60. The patient's overall survival from the initial diagnosis was 590 days (approximately 19.5 months).

## Discussion

The most common explanation for the identification of multiple intracranial tumors in patients is metastatic cancer. However, multiple glioblastomas occur in only a few percent of cases [5]. These may present as multifocal or multicentric diseases, as in our patient, who had two distinct tumors in opposite hemispheres. Additionally, considerable intratumoral genetic heterogeneity has been demonstrated even within a single glioblastoma [9]. This inherent diversity further complicates the question of whether multiple spatially separated lesions in a patient arise from a single clonal origin or develop independently.

Recent studies have focused on the molecular characteristics of multifocal glioblastomas to better understand their pathogenesis. A comprehensive analysis of six cases of multifocal glioblastoma by Abou-El-Ardat et al. demonstrated a monoclonal origin of spatially separated lesions, with all tumor samples from each patient sharing key early genetic alterations [10]. In that study, all 12 tumors (from six patients) harbored TERTp mutations and deletions of chromosome 10 (including the loss of the phosphatase and tensin homolog (PTEN) tumor suppressor gene), and recurrent alterations in EGFR, PDGFRA, and CDKN2A/B were frequently observed across lesions in the same patient [10]. These findings suggest that certain driver mutations, such as TERTp mutations and chromosome 10 loss, occur early in gliomagenesis and are retained as a common clonal signature in multifocal diseases.

Indeed, TERTp mutations are known to be early events in the development of glioma and other cancers [11,12]. Consistent with this, a recent study reported that TERTp-mutant, IDH-wildtype glioblastomas have a higher propensity for multifocal or multicentric presentation, a more invasive growth pattern, and a poorer prognosis than TERTp-wildtype tumors [13]. Furthermore, the epigenetic upregulation of telomerase may play a role in tumor aggressiveness, and hypermethylation of TERTp can increase TERT expression, which has been implicated in glioblastoma progression [14]. Additionally, alterations in growth factor signaling pathways may predispose patients to multifocal tumor growth. For example, PDGFRA gene mutations, which are present in approximately 20-25% of GBMs, have been associated with a significantly higher incidence of multifocal disease [15].

In the present case, both tumors shared a TERTp mutation, supporting the notion that* *TERTp activation was an initiating molecular event. This raises the possibility that, although not proven in a single case, the two spatially separated lesions arose from a common clonal origin before diverging. However, each lesion contained additional distinct mutations. The right temporal lobe tumor exhibited EGFR amplification and homozygous deletion of CDKN2A/B, and genetic changes have often been linked to more aggressive tumor behavior. These alterations may have contributed to the rapid recurrence of the right-sided lesion. In contrast, the left temporal lobe tumor harbored a BRAF p.Val600Glu (V600E)*​​*​​​​​mutation, which is a relatively rare event in adult glioblastomas, occurring in approximately 2-3% of cases. The BRAF p.Val600Glu (V600E)*​​​​​*​​ mutation has been reported to reduce responsiveness to standard chemotherapy (e.g., temozolomide) but also creates an opportunity for targeted therapy with BRAF and mitogen-activated protein kinase (MEK) inhibitors [16].

Clinically, gliomas with BRAF p.Val600Glu (V600E) mutations have shown responsiveness to molecularly targeted treatments, as demonstrated in trials for pediatric gliomas [15], and may be associated with a slightly better prognosis in certain contexts [17]. There is also a distinctive histopathological correlation: tumor cells carrying the BRAF p.Val600Glu (V600E) mutation often exhibit markedly enlarged, pleomorphic nuclei [18]. Consistent with this, the left-sided tumor in our patient with the BRAF mutation displayed more giant cell features on microscopy, whereas the right-sided tumor did not. Notably, after one year of follow-up, the tumor without the BRAF mutation (right side) recurred, whereas the BRAF-mutant left tumor remained in remission. Interestingly, while the BRAF p.Val600Glu (V600E) mutation has been associated with reduced sensitivity to temozolomide in some contexts, our patient's BRAF-mutant tumor did not recur following standard chemoradiotherapy. This paradoxical finding warrants further consideration. Taken together, these observations highlight the clear clinical implications of the molecular divergence between the two lesions. Distinct genetic alterations may lead to different tumor behaviors, treatment responses, and recurrence patterns even within the same patient. This underscores the necessity of performing lesion-specific molecular profiling in multicentric glioblastoma to guide individualized treatment strategies. To the best of our knowledge, the BRAF p.Val600Glu (V600E)*​​​​*​​​ mutation has rarely been reported in multicentric glioblastomas, making this case unique. This suggests that even in highly aggressive conditions, such as multifocal glioblastoma, the presence of certain mutations, such as BRAF p.Val600Glu (V600E)*​​​*​​​​, could favorably influence tumor behavior and open the door to personalized treatment options.

## Conclusions

This case contributes rare molecular evidence supporting divergent clonal evolution in multicentric glioblastoma. This case highlights the remarkable intra-patient molecular heterogeneity that can occur in multicentric glioblastomas. Two anatomically distinct glioblastomas in the same patient shared a common TERTp mutation yet exhibited divergent molecular profiles and clinical behaviors. Such molecular discordance between lesions poses a major challenge for precision medicine, as a therapy effective for the genetic makeup of one lesion may not be optimal for another. Comprehensive genetic profiling of each tumor lesion, when clinically feasible, can provide critical information to guide individualized treatment strategies. The accumulation and systematic analysis of similar cases are warranted to deepen our understanding of the clonal evolution, pathogenesis, and optimal management of multicentric glioblastoma.
